# Stromal alterations in ovarian cancers via wavelength dependent Second Harmonic Generation microscopy and optical scattering

**DOI:** 10.1186/s12885-017-3090-2

**Published:** 2017-02-06

**Authors:** Karissa B. Tilbury, Kirby R. Campbell, Kevin W. Eliceiri, Sana M. Salih, Manish Patankar, Paul J. Campagnola

**Affiliations:** 1 Laboratory for Optical and Computational Instrumentation, Department of Biomedical Engineering, University of Wisconsin – Madison, 1550 Engineering Drive, Madison, WI 53706 USA; 2 Medical Physics Department, University of Wisconsin – Madison, 1111 Highland Avenue, Madison, WI 53706 USA; 3 Morgridge Institute for Research, 330 N. Orchard Street, Madison, WI 53715 USA; 4 Department of Obstetrics and Gynecology, University of Wisconsin – Madison, 600 Highland Avenue, Madison, WI 53706 USA

**Keywords:** Ovarian cancer, Second Harmonic Generation (SHG) imaging microscopy, Optical scattering, Extracellular matrix (ECM)

## Abstract

**Background:**

Ovarian cancer remains the most deadly gynecological cancer with a poor aggregate survival rate; however, the specific rates are highly dependent on the stage of the disease upon diagnosis. Current screening and imaging tools are insufficient to detect early lesions and are not capable of differentiating the subtypes of ovarian cancer that may benefit from specific treatments.

**Method:**

As an alternative to current screening and imaging tools, we utilized wavelength dependent collagen-specific Second Harmonic Generation (SHG) imaging microscopy and optical scattering measurements to probe the structural differences in the extracellular matrix (ECM) of normal stroma, benign tumors, endometrioid tumors, and low and high-grade serous tumors.

**Results:**

The SHG signatures of the emission directionality and conversion efficiency as well as the optical scattering are related to the organization of collagen on the sub-micron size scale and encode structural information. The wavelength dependence of these readouts adds additional characterization of the size and distribution of collagen fibrils/fibers relative to the interrogating wavelengths. We found a strong wavelength dependence of these metrics that are related to significant structural differences in the collagen organization and are consistent with the dualistic classification of type I and II serous tumors. Moreover, type I endometrioid tumors have strongly differing ECM architecture than the serous malignancies. The SHG metrics and optical scattering measurements were used to form a linear discriminant model to classify the tissues, and we obtained high accuracy (>90%) between high-grade serous tumors from the other tissue types. High-grade serous tumors account for ~70% of ovarian cancers, and this delineation has potential clinical applications in terms of supplementing histological analysis, understanding the etiology, as well as development of an in vivo screening tool.

**Conclusions:**

SHG and optical scattering measurements provide sub-resolution information and when combined provide superior diagnostic power over clinical imaging modalities. Additionally the measurements are able to delineate the different subtypes of ovarian cancer and may potentially assist in treatment protocols. Understanding the altered collagen assembly can supplement histological analysis and provide new insight into the etiology. These methods could become an in vivo screening tool for earlier detection which is important since ovarian malignancies can metastasize while undetectable by current clinical imaging resolution.

## Background

Ovarian cancer remains the most deadly gynecological cancer among women with an aggregate 5-year survival rate of ~45%. However, the specific rates are highly dependent on the stage of the disease upon diagnosis. For example, diseased localized to the ovary has a 5-year survival rate of ~92%, whereas this decreases precipitously to 27% for metastatic disease. With current methods only 15% percent of the patients are diagnosed at stage I (American Cancer Society Cancer Facts and Figure 2015). Early detection is difficult due to vague symptoms (e.g. bloating, abdominal discomfort) as well as the lack of effective clinical screening/imaging tests. Although the CA125 tumor marker and trans vaginal ultrasound (TVUS) have been investigated as screening strategies, both of these methods are not sufficiently selective or specific to be employed as clinical diagnostic tests for early detection of ovarian cancer [1, 2]. For example, many patients who had frequent screening involving CA-125 blood serum levels and TVUS had already developed widespread high grade ovarian cancer when positively diagnosed [3].

Recently, genetic analysis has led to identification of several subtypes of ovarian tumors [4, 5]. Type I tumors include borderline, mucinous, low-grade serous (LGS), and endometrioid cancers. High-grade serous (HGS) ovarian tumors, which are the predominant type of cancer detected in patients, are classified as Type II tumors. This emerging understanding of classification of ovarian cancer is also leading to the realization that subtype-specific strategies need to be developed for efficient treatment of the malignancy. Therefore, it is not only important for a diagnostic test to detect the presence of ovarian cancer but also to classify the specific subtype of the disease [6, 7]. No current diagnostic method is able to meet these two criteria.

Examination of remodeling of the extracellular matrix (ECM) through higher resolution and highly specific approaches holds great promise in helping to address these diagnostic needs. This is because these alterations are thought to be a critical step in the initiation and progression of many epithelial carcinomas [8]. These alterations can be in the form of increased collagen content (desmoplasia), modified morphology (e.g. collagen fiber alignment) or up-regulation of different collagen isoforms [9]. Screening techniques that could quantitate changes in the collagen remodeling of the ECM could reveal early changes in architecture which could be used to help detect, identify, and stage disease in patient samples.

To investigate this possibility, we implemented high resolution optical microscopy and spectroscopy tools to quantify changes in the ECM across a spectrum of human ovarian cancers. We use Second Harmonic Generation (SHG) imaging microscopy [10] to objectively quantify differences in ECM structure of normal stromal, type I and II tumors, and also benign lesions. SHG is a coherent process in which two photons are up-converted to exactly twice the frequency (half the wavelength) of an excitation laser. The contrast mechanism of SHG results from a nonlinear polarization given by: *P* = *χ* ^(2)^ *EE* where P is the induced polarization, E is the electric field vector of the laser, and χ^(2)^ is the second-order nonlinear susceptibility tensor. The nonlinear susceptibility tensor χ^(2)^ dictates the intensity of the SHG signal and requires a non-centrosymmetric assembly of harmonophores, which have permanent dipole moments on the size scale of λ_SHG_, to be nonvanishing. This technique is collagen specific and permits imaging deep into tissues (few hundred microns) with intrinsic optical sectioning [11]. The underlying physics permits probing collagen architecture from the macromolecular, supramolecular, and fibril levels through the fiber levels of organization [10].

SHG microscopy has been used in previous studies to investigate the alterations of the stroma in human and mouse models in ovarian cancer using image analysis approaches that interrogated the fiber alignment [12–15]. We previously developed and utilized a more generalizable approach based on the underlying SHG creation physics to differentiate the collagen organization and applied the method to compare HGS tumors and normal stroma [16]. SHG is a coherent process and is dependent on phasematching, Δ*k* = *k*_2*ω*_ − 2*k*_*ω*_ = 0, (where k_2ω_ and k_ω_ are the wave vectors for the SHG and incident photon, respectively). Biological phasematching conditions are not ideal (i.e. Δ*k* ≠ 0), and as a result of conservation of momentum, there is a specific emission pattern (i.e.forward (F_SHG_) and backward (B_SHG_) components) that depends on the tissue structure. We thus utilize the intensity ratio, F_SHG_/B_SHG_, as a metric that arises from the fibril size and packing relative to the SHG wavelength, λ_SHG_. Phasematching also has implications on the observed SHG intensity, where the relative SHG intensity scales as sin(mΔkL/2) where *m* is an integer and thus becomes less efficent for larger phase mismatch, i.e. larger Δk values, which correspond to more random structures compared to the length scale of λ_SHG_ [17]. For normal and HGS tumor stroma, we extracted different F_SHG_/B_SHG_ values, that based on a mathematical model we developed [17], were consistent with TEM images of the respective collagen fibrils [16]. These differences in structure also led to quantifiable SHG intensity differences between these tissues, where the intensity depends on both the collagen concentration and organization. We further utilized measurements of optical scattering in combination with the SHG metrics. Scattering measurements are also sensitive to ECM architecture and have proven to be highly capable of delineating cancer from normal tissue in many organs [18, 19].

We now extend those earlier efforts and perform these measurements and analysis for several tumor types (normal, benign, LGS Type I, endometrioid Type I, and HGS Type II tissues) and also across a large excitation wavelength range (780–1160 nm). We will show the wavelength dependencies encode structural information that identifies changes in collagen fibril/fiber assemblies. All the SHG and optical scattering metrics are reflective of the size and distribution of ECM components relative to the interrogating wavelength. These studies on ECM alterations will lead to better insight into ovarian cancer etiology and progression.

## Methods

### Tissue removal and preparation

Malignant ovarian tissues were obtained using an IRB approved protocol from consented patients undergoing surgical de-bulking treatment for ovarian cancer. Normal ovaries were obtained from consented patients (aged 45–65) undergoing bilateral salpingo-oophorectomy for benign neoplasms, fibroids, endometriosis, and uterine prolapse. All tissues were immediately fixed in 4% formalin and refrigerated for 24 h and then switched to phosphate buffered saline (PBS). The specimens were sectioned *en face* using a Leica Vibratome 1200S (Leica Biosystems, Buffalo Grove, IL) to thickness of 50 μm and 100–150 μm for optical scattering measurements and SHG imaging studies, respectively. Tissues were classified by a gynecological pathologist into normal (*n* = 4), benign (*n* = 4), borderline/low-grade serous Type I (*n* = 4), endometrioid Type I (*n* = 3), and high-grade serous Type II (*n* = 3). The genomic mutations of the tissues are not known.

### SHG microscopy

#### SHG imaging system

The essentials of the SHG imaging system has been described elsewhere [10]. A femtosecond laser was coupled to a home-built laser-scanning system (WiscScan; http://loci.wisc.edu/software/wiscscan) on a fixed stage upright microscope base (BX61WI, Olympus, Center Valley, PA) microscope. A 40 × 0.8 numerical aperture (NA) water immersion objective and a 0.9 NA condenser are used for excitation and collection, respectively, of the forward SHG signal providing lateral and axial resolutions of approximately 0.7 and 2.5 μm, respectively. The backward SHG was collected in a non-descanned geometry, where the detector was in the infinity space. Both detectors are H7422-40P GaAsP photomultiplier tubes (Hamamatsu, Hamamatsu, Japan). Calibration of the forward and backward detection pathways was performed using the two-photon excited fluorescence imaging of beads emitting in the same wavelength range as the collected SHG signal. The laser excitation range was 780–1160 nm provided by Chameleon Ultra Ti:Sapphire oscillator and a synchronously pumped APE Optical Parametric Oscillator (Coherent, Santa Clara, CA). For all excitations, the SHG signal was isolated with 20 nm full width half maximum bandpass filters centered at the corresponding SHG wavelength (Semrock, Rochester, NY). Circularly polarized light, determined at the focus, was used throughout to equally excite all fiber orientations [10].

#### 3D SHG imaging

Depth dependent Forward/Backward (F/B) measurements were made for the entire thickness of the ovarian sections in three different locations, from which the data was obtained sequentially across the wavelength range. The SHG intensities were integrated across the whole fields of view using both FIJI (an open-source ImageJ platform for image analysis) [20] and MATLAB (MathWorks, Natick, MA). The measured forward attenuation, i.e. the rate of SHG intensity decrease with increasing depth into tissue, was also used in characterizing tissue alterations. Due to intrinsic heterogeneity in concentration of biological tissues, we found it necessary to normalize the SHG intensity response to account for local variability within the same tissue (different fields of view) and to make relative comparisons between tissues. Normalization of each optical section within each optical series was self-normalized with the average maximum intensity value. The normalized forward attenuation and the F/B data were taken concurrently. 3D renderings were performed in Imaris (Bitplane AG, Zurich, Switzerland).

#### Monte Carlo simulations

The SHG directional emission ratio, F_SHG_/B_SHG_, and relative SHG conversion efficiency were decoupled from the depth dependent SHG response curves using Monte Carlo simulations based on an adapted MCML (Monte Carlo Multi-Layer) [21] framework [22, 23]. The F_SHG_/B_SHG_ at each wavelength was extracted by running a series of forward simulations of the measured forward/backward vs depth curve based on the corresponding measured optical properties (described below) and initial guesses of the emission directionality and then obtaining the best fit to the simulations. The relative SHG conversion efficiency at each excitation wavelength was determined by modeling the normalized forward SHG attenuation response using a similar simulation incorporating the extracted F_SHG_/B_SHG_ and wavelength specific optical parameters at both the excitation and SHG wavelengths. All forward simulations were completed using parallel computing at the Wisconsin Center for High Throughput Computing at the University of Wisconsin-Madison and the values were stored in tables, permitting all the image processing to be performed in custom MATLAB scripts.

### Optical scattering measurements

We determined the optical properties of tissue through an average of three independent locations within a tissue specimen, where these parameters include the refractive index *n,* absorption coefficient *μ*_*a*_, scattering coefficient *μ*_*s*_, and scattering anisotropy *g*. An estimate of the bulk refractive index was determined by a total internal reflection measurement of the sample as described by Li et al. [24]. The absorption coefficient is negligible in most fibrillar tissues as *μ*_*a*_ < < *μ*_*s*_ and was previously confirmed in ovarian tissues [16].

The scattering coefficient and anisotropy were obtained independently in a multi-step process using on-axis attenuation measurements which is applicable when *μ*_*a*_ < < *μ*_*s*_ [25]. These values were measured at 390, 445, 494, 535, 780, 890, 988, and 1070 nm using the tunable Ti: Sapphire laser tunable and an external BBO frequency doubling crystal. SHG imaging was used to determine the tissue thickness to obtain *μ*_*s*_ from the Beer-Lambert law. We then report the reduced scattering coefficient, *μ*_*s*_′, which is a merged property defined by:

1 $$ {\mu}_s^{\prime }={\mu}_s\left(1- g\right) $$

### Statistical analysis

The canonical variable linear discriminant and logistic progression were performed in SAS (SAS Institute Inc., Chicago, IL) using the CANDISC procedure, where this was applied to all sample data points. Using the canonical variable weights, a model based on the relative SHG conversion efficiency, F_SHG_/B_SHG_, *μ*_*s*_, and their wavelength dependence was used to classify the images. ROC curves were computed by comparing the modeled response versus the true or assigned tissue classification via a logistic regression. All the ANOVA and Fisher LSD statistical tests were performed in Origin 9.1 (OriginLab, Northampton, MA). For the latter, *p* < 0.05 was considered significant.

## Results

### Fiber morphology

**Fig. 1:**
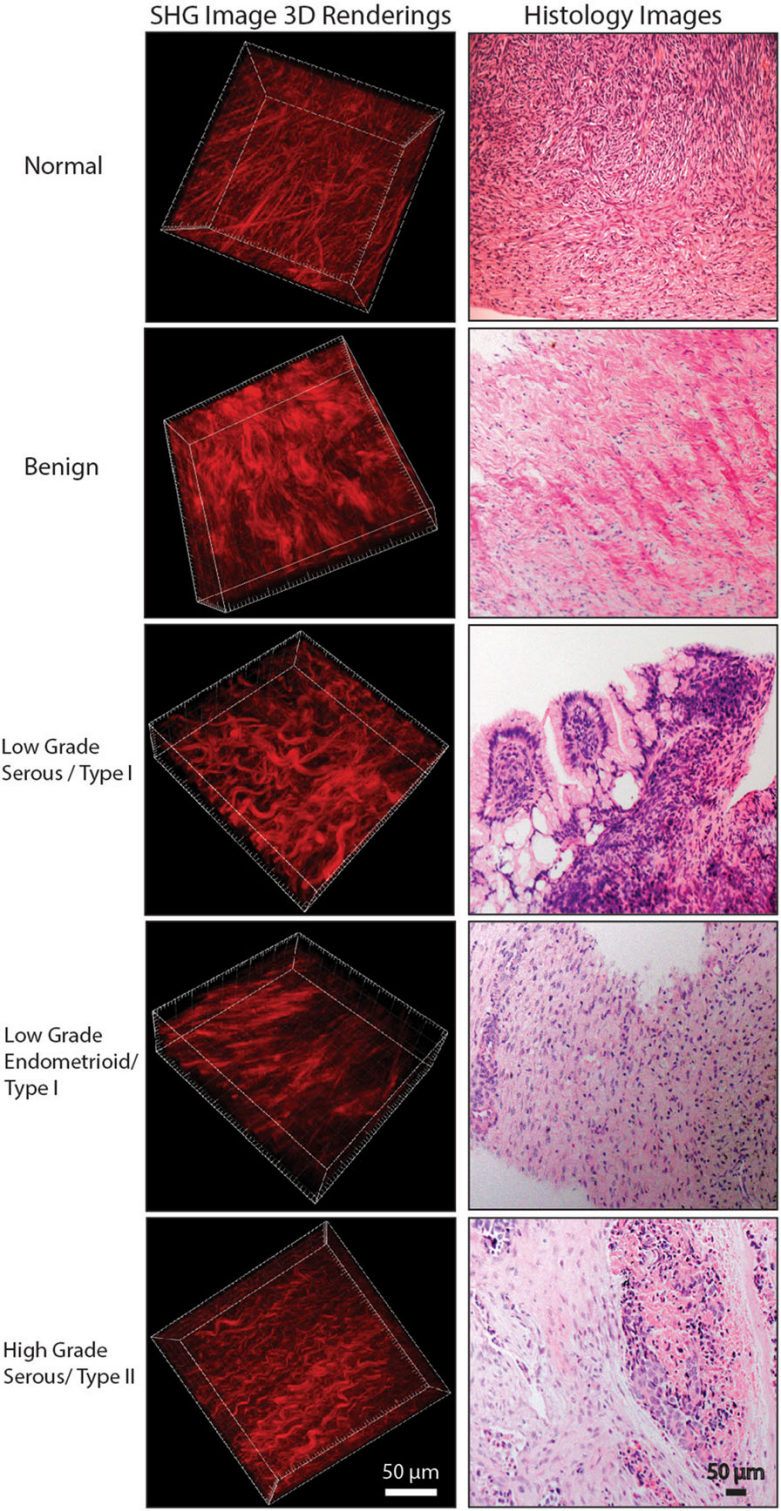
Left column shows 3D renderings of forward directed SHG images of representative normal stroma, benign, LGS, endometrioid, and HGS ovarian tumors obtained at 890 nm excitation. The tissue sections were ~100 μm in thickness. Right column is representative H&E staining of the same tissue. Scale bar = 50 μm

The left and right columns of Fig. 1 show a representative 3D SHG rendering and H&E histology image of each tissue type studied in this paper. Normal post-menopausal ovarian tissues have a loose-mesh like collagen network, where the “holes” in the images correspond to stromal fibroblasts, which are transparent in SHG contrast. Malignant tissues are heterogeneous in nature; however, HGS tissue morphology is highly conserved within the patient population displaying a dense, highly aligned network of long wavy collagen fibers. LGS tissues are fibrotic with tightly packed shorter collagen fibers whereas endometrioid tissues have low collagen density and highly aligned thin collagen fibers. Benign tissues are fibrotic with wavy networks of large collagen fibers. We note that there is not significant variation in the collagen morphology across the different sampling regions as we limit the analysis s to collage rich areas near the surface epithelium. These overall patterns in each case are also observed in the corresponding H&E sections visualizing the collagen and cell nuclei.

It is difficult to precisely determine fiber diameters as they are not all discrete and often overlap within the resolution of the microscope. However, we approximated the diameters by thresholding and measuring the thickness by creating a profile across several discretized fibers. We then found average values (standard error in parenthesis) of: normal: 2.7 (0.1) μm; benign: 3.6 (0.2) μm; endometrioid: 1.9 (0.1) μm; LGS: 3.4 (0.2) μm and HGS: 2.3 (0.1) μm. The diameters of the LGS and benign were similar; those of the endometrioid and HGS were similar, but all the others were different from each other.

### Tissue scattering properties

The wavelength dependence of the reduced scattering coefficient, *μ*_*s*_′, depends on the spatial distribution of the refractive index due to structural differences on size scales smaller than the diffraction limit. For rigorous tissue characterization, Backman et al. used the flexible Whittle-Matérn correlation function to quantify the distribution of length scales where the output is the shape factor m, which corresponds to one half of the fractal dimension [26, 27]. The shape factor is connected to the spectral dependence of the reduced scattering coefficient through the power law expression by: μ_s_^′^(λ) ~ λ^(2m-4)^ where higher *m* values are associated with larger, more ordered structures on the approximate size scale of 50 nm to 1 μm.

**Fig. 2:**
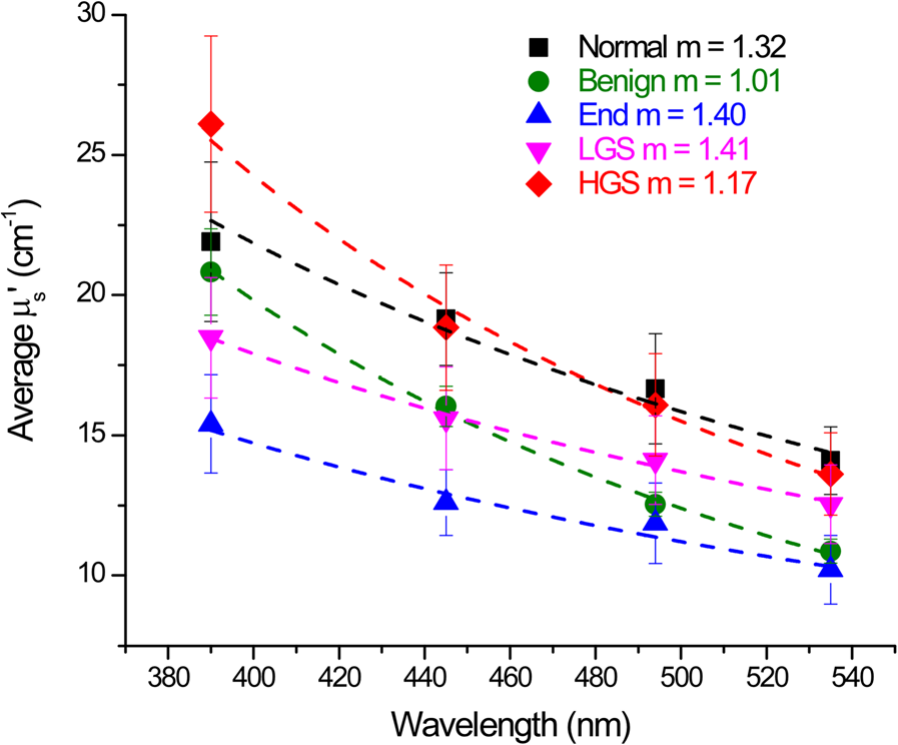
Wavelength dependence of the reduced scattering coefficient *μ*_*s*_′ over the wavelength range used for SHG imaging (390–535 nm) for the normal stroma and ovarian tumors. The best fit to the scattering power law from the corresponding *m* factor is shown with the experimental data. All curves are the average response from normal *n* = 4; benign *n* = 4; endometrioid *n* = 3; LGS *n* = 4; and HGS *n* = 3. Error bars are standard error

We measured the spectral dependence of the reduced scattering coefficient, *μ*_*s*_′, for the five tissue types over the range of 390–535 nm (corresponding to the wavelengths where the SHG metrics were probed) and the results are shown in Fig. 2. The spectral dependencies of the normal and malignant ovarian tissues are different. HGS ECM had the greatest scattering intensity, indicating increased tissue density. This is consistent with the dense fibrillar structure seen in the 3D rendering in Fig. 1. The endometrioid tumors had the weakest absolute scattering, which is also consistent with the SHG images showing sparse (but regular) fibers. We stress that the measured *μ*_*s*_′ values reflect both the fiber and cellular components, however the former is much more strongly scattering. The other tissues (LGS, normal and benign) showed intermediate behavior. These were mostly statistically different at 390 nm (*p* < 0.05), and some were significant at other wavelengths. In addition to the absolute scattering intensities, we also compared the tissues through the fitted *m* values. By this metric, LGS and endometrioid tissues are the most ordered with *m* values of 1.41 and 1.40, respectively, where these were not distinct; however, the *μ*_*s*_′ were different at 390 and 445 nm. Benign tissues (*m* = 1.01) show the least order, followed by HGS (*m* = 1.17), then normal tissues (*m* = 1.32). Given that both *μ*_*s*_′ and *m* are different for some of the tissues, we concluded that both the scattering intensity and spectral dependence are needed to describe the tissues fully and provide the best differentiation. We note that this is not unexpected as the *m* values correspond to organization on the ~50 nm-1 μm size scale, while *μ*_*s*_′ is a merged parameter reflecting both organization (g) and density (*μ*_*s*_). Thus, higher values of both parameters may not occur for a single tissue.

### Wavelength dependence of F_SHG_/B_SHG_

The F_SHG_/B_SHG_ metric is a subresolution parameter that arises from the fibril size and packing relative to λ_SHG_ [17]. The wavelength dependence of this parameter can yield further discrimination than possible by single wavelength measurements as tissues having differing fibril architecture relative to the SHG wavelengths will have a different wavelength dependent F_SHG_/B_SHG_. Here we utilize a broad excitation range (780–1160 nm), which is limited by optical transmission of the SHG (390 nm) and excitation (1160 nm) wavelengths and then extract F_SHG_/B_SHG_ as described in the Methods section of this manuscript.

**Fig. 3:**
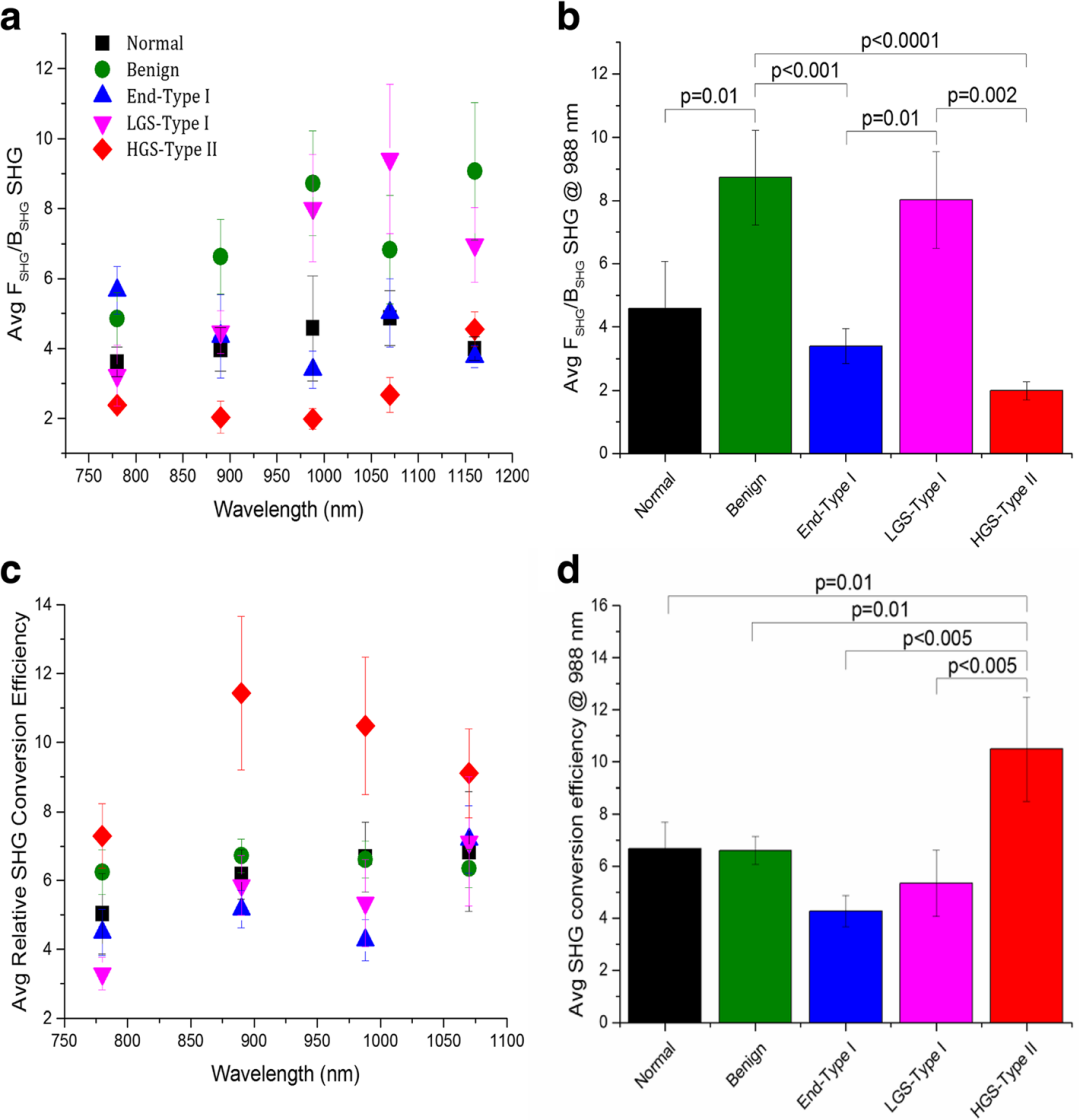
Extracted F_SHG_/B_SHG_ emission directionality and SHG conversion efficiencies of the ovarian tissues obtained via Monte Carlo simulations. **a** Wavelength dependent F_SHG_/B_SHG_ emission directionality response from 780 to 1160 nm (excitation wavelengths) and **b** Average F_SHG_/B_SHG_ emission directionality at 988 nm, where best delineation between the tissues was obtained; p values showing significant differences are indicated. **c** Wavelength dependent SHG conversion efficiency response from 780 to 1070 nm excitation wavelengths. **d** Relative conversion efficiencies at 988 nm, where best delineation between tissues was obtained; p values showing significant differences are indicated. All curves are the average response from normal *n* = 4; benign *n* = 4, endometrioid *n *= 3; LGS *n* = 4; and HGS *n* = 3. Error bars are standard error

The data are summarized in Fig. 3a. The optimized simulation in each case was not statistically different (via χ^(2)^ test) from the measured data, implying a good fit to F_SHG_/B_SHG_. All ovarian tissue types displayed an increase in F_SHG_/B_SHG_ emission directionality with increasing wavelength. This result is expected due to phasematching considerations, as we have reported for other tissues [23]. This arises because the dispersion in refractive index between the laser excitation and SHG wavelengths decreases at longer wavelengths, decreasing the phase mismatch efficiency and increasing the forward SHG component [17].

However, the form of the wavelength dependent increase in F_SHG_/B_SHG_ is tissue specific. HGS tumors have the smallest F_SHG_/B_SHG_. One physical scenario that gives rise to a low F_SHG_/B_SHG_ is when the fibril size and spacing are regular and much smaller than λ_SHG_ where this results in efficient quasi-phasematching (QPM), thus increasing the B_SHG_ emission [17, 28]. We previously verified this through TEM imaging of HGS tumors [16]. The higher emission directionality for the LGS ECM implies that the collagen fibrils are larger and more disorganized than those in both normal and HGS tissues. Overall, benign serous tissues have the highest F_SHG_/B_SHG_ emission directionality indicating this tissue type has the largest collagen fibrils. This is also suggested by this tissue having the largest measured fibers from Fig. 1. Normal tissues have intermediate F_SHG_/B_SHG_ values between HGS and LGS tissues, demonstrating that both these tissues have altered collagen assembly than normal post-menopausal ovarian stroma. However, the alterations result in opposite trends, suggesting the modifications have different forms. This is consistent with type I and type II classes representing genetically different diseases [4].

The optimal single wavelength to differentiate the tissue types by the F_SHG_/B_SHG_ metric was 988 nm. The F_SHG_/B_SHG_ values and statistical analysis at this wavelength are shown in Fig. 3b. The F_SHG_/B_SHG_ statistically differentiates the collagen fibril size and assembly of HGS tissues from LGS tissues and also from benign tissues (*p* ≤ 0.002) despite small samples sizes of 3 or 4 patients per tissue type. Benign tissues are also statistically different (*p* ≤ 0.01) from normal and endometrioid tissues. Also interesting is the difference in collagen fibril assembly of LGS and the endometrioid tissues (*p* = 0.01) highlighting the heterogeneity of collagen features within these tissues, even though both are classified as Type I [5]. Although 988 nm excitation, provided statistical distinction in the collagen fibril assembly between different types of ovarian cancer, this metric by itself was not able to provide necessary separation between normal and LGS and normal from HGS tumors. However, these were differentiated by including this metric in the multivariate analysis to be shown later.

### SHG conversion efficiency

We also determined the wavelength dependence of the relative SHG conversion efficiency as a means to characterize the tissue structure. The conversion efficiency is a coupled effect of the collagen concentration (square thereof) and its fibrillar organization on the size scale of λ_SHG_ [16, 22]. Monte Carlo simulations using the measured optical properties at the fundamental and SHG wavelengths extracted the SHG conversion efficiency by obtaining the best fit to the conversion efficiency, in analogy with obtaining the F_SHG_/B_SHG_ values.

The extracted SHG conversion efficiencies for the five tissue types across the 780–1070 nm range are shown in Fig. 3c. As absolute values of the conversion efficiency are not readily obtainable, we plot them normalized to the maximum intensity (here HGS at 890 nm). Overall, the HGS tissues have the most efficient SHG relative to the normal and other ovarian cancer subtypes, indicating the highest collagen concentration and/or organization. We note that these properties are not separable by SHG imaging. Optimal values of relative SHG conversion efficiency were found at 988 nm. Here, the HGS tissues were statistically different (*p* ≤ 0.01) from normal and all other types of ovarian cancer. LGS, endometrioid, benign, and normal tissues had similar values of relative SHG conversion efficiency; however, the wavelength dependent response is slightly different. LGS tissues have the greatest wavelength dependent increase in the SHG conversion. We note that the HGS conversion efficiency decreases with wavelength, whereas the other tissues show an increase, suggesting large structural differences which will be addressed in the Discussion.

### Multi-variable linear discriminant analysis

In this study, three sub-optical resolution properties were obtained to further understand the ECM remodeling associated with ovarian cancer. Combining these independent metrics provides a more robust characterization of the normal and diseased ovarian stroma. This is important as not all parameters at all wavelengths were statistically different. To this end, we used a linear discriminant with three canonical variables (see methods), where the results are summarized in Table 1. All the individual cells in Table 1 are pairwise comparisons between the tissue types, as opposed to one versus the rest classification. These metrics successfully classified HGS from normal, benign, LGS, and endometrioid tissues with excellent accuracy indicating that this multi-variable approach has high fidelity in describing the altered ECM architecture. Normal stroma was moderately differentiated from benign, LGS, and endometrioid with accuracies between 75 and 80%. Even this moderate differentiation is comparable to the collective diagnostic accuracy of clinical imaging modalities. LGS and benign tumors had the poorest differentiation (~65%) which is expected as these tissues had the most similar morphology (Fig. 1) [29].

**Table 1:** Classification accuracy of ovarian tissues based on the multi-parameter canonical linear discriminant model

	High-grade serous (*n* = 3)	Benign (*n* = 4)	Low-grade serous (*n* = 4)	Endometrioid (*n* = 3)
Normal (*n* = 4)	89.6%	77.6%	77.8%	79.3%
Benign (*n* = 4)	93.7%			
Endometrioid (*n* = 3)	95.6%	76.6%	69.7%	
Low-grade serous (*n* = 4)	96.9%	67.3%		

## Discussion

We previously developed a heuristic model based on phasematching between the excitation and SHG waves that correlates collagen fibril size and assembly with the F_SHG_/B_SHG_ emission directionality [17]. In this context, we defined a “domain,” meaning either a single fibril/fiber or smaller fibrils overlapped together to achieve the same diameter. We showed that larger domains on the size scale of λ_SHG_ produce larger F_SHG_/B_SHG_, where smaller sizes yield lower values, and also weaker SHG intensities. A special case can arise, when the fibrils are much smaller than λ_SHG_ and equally spaced, through QPM, producing relatively more efficient backward SHG.

**Fig. 4:**
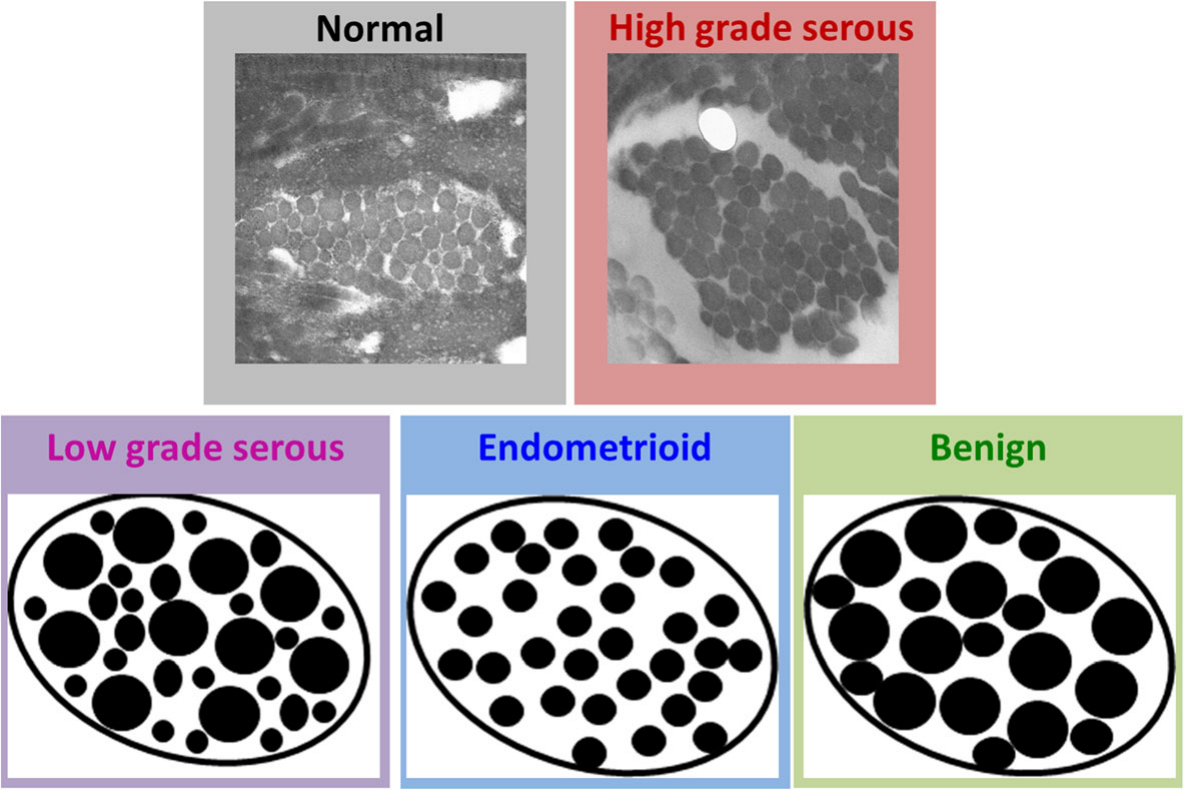
Collagen fibril assembly based on the wavelength dependent phasematching response. TEM images of normal and HGS ovarian tissues. Cartoons of the LGS, endometrioid, and benign ovarian tissues

The phasematching arising from the distribution of fibril sizes is always relative to λ_SHG_; thus, if the tissues have different distributions of domain sizes, this will result in a different wavelength dependence of the F_SHG_/B_SHG_ parameter. The overall increase in F_SHG_/B_SHG_ with increasing wavelength is expected by simple phasematching conditions based on the wavelength dependence of the refractive index [23]. The other factor of this increase is the extent the domain matches the size scale of λ_SHG._ The HGS tissues show the weakest wavelength dependence, and this is consistent with our previously reported TEM images, which showed the fibrils were all ~60 nm in diameter [16]. Physically this would correspond to near single value of the phase mismatch and should result in little wavelength dependence as the fibrillar domain is also much smaller than λ_SHG._ The low F_SHG_/B_SHG_ is further consistent with arising from the QPM mechanism. While we do not have TEM images for the LGS and benign tissues, within the resolution of the microscope (Fig. 1), these fibers are larger than the HGS and normal stroma and have larger F_SHG_/B_SHG_ values and have a more pronounced wavelength dependence. The large wavelength dependent increase was observed for the LGS tumors, indicating a broader distribution of fibril sizes. Similar to the HGS tumors, the endometrioid tumors have a weak wavelength dependence of F_SHG_/B_SHG_ values and also narrow distribution of fibers sizes as seen in Fig. 1. In sum, this analysis determining the wavelength dependence of F_SHG_/B_SHG_ values affords obtaining sub-resolution structural information of the fibril size and packing in intact tissues without performing TEM analysis as seen in Fig. 4.

Differences in collagen density and organization are also manifested in the relative SHG conversion efficiencies. The SHG conversion efficiency is based both on the magnitude of nonlinear susceptibility tensor χ^(2)^ which is a property of the collagen itself, and phasematching, which arises from the organization relative to λ_SHG_ [17]. We recently reported the wavelength dependence of the SHG in murine tendon, and showed a sharp decrease in conversion efficiency (~5 fold) over the same wavelength range used here. This decrease was ascribed mostly to decreasing χ^(2)^ rather than the improved phasematching at longer wavelengths [30]. Furthermore, this was physically reasonable as the diameters of murine tendon fibrils are narrowly distributed [31], and as discussed above, this leads to a weak wavelength dependence of phasematching as well as a small change in the concomitant F_SHG_/B_SHG_ values. Here, we performed this analysis on the different ovarian ECM tissues and compared their relative conversion efficiencies and wavelength dependence.

As shown in Fig. 3c, the HGS tissues had the highest SHG conversion efficiency. This result was expected based on the appearance of the collagen morphology in Fig. 1 and strongest scattering (due to highest density) in Fig. 2. We also point out that the HGS tissues had the opposite dependence on wavelength, where the relative conversion efficiency decreased instead of the increase with longer wavelengths seen from the other tissues. The HGS response is quite similar to that observed for tendon in our prior work [30], where the χ^(2)^ component dominates the increased phasematching at longer wavelengths. This is further consistent with the lower F_SHG_/B_SHG_ emission directionality shown in Fig. 3a and implies the fibril structure is small relative to λ_SHG_ with a narrow distribution of diameters. The relative SHG conversion efficiencies were similar at 988 nm (like the F_SHG_/B_SHG_ values). All the other tissue types showed a slight increase with wavelength. From a structural perspective, this implies the fibril domain is larger than the HGS tissues and is also characterized by a larger distribution of fibril sizes (Fig. 4). Thus, while arising from different attributes, the emission directionality and relative conversion efficiencies both yield consistent structural information. Importantly, these structural aspects arise from features below the resolution limit of the microscope but are obtainable due to the physical underpinnings of the SHG process.

We also characterized the tissue architecture using optical scattering measurements of both the scattering intensity and its wavelength dependence (Fig. 2) [18, 19, 26]. The μ_s_′ and fit *m* values were obtained over the SHG wavelength range and good separation was achieved between most of the tissue types. We also performed this fit across the entire spectral range that included all the excitation and SHG wavelengths used in the SHG analyses. This approach yielded almost no discrimination between the tissues, where the reduced scattering coefficients at the longer wavelengths (>780 nm) were largely similar. In analogy to the SHG data, the scattering coefficients are also related to the scatterer size relative to the interrogating wavelength. The higher sensitivity at the shorter wavelengths implies that the scattering structures lie within this size range. Although optical scattering and SHG arise from different physics, we found similar size scales are operative in both cases. The data identified the most sensitive wavelength range to use for diagnostic purposes for both SHG and optical scattering.

Although the individual metrics, F_SHG_/B_SHG_ emission directionality, relative SHG conversion efficiency, *μ*_*s*_^*′*^, and their respective wavelength dependence provided differentiation in some cases, each metric described only a single feature of the ovarian ECM. Combination of the metrics in a canonical linear discriminant provided enhanced classification based on the weights of the three individual variables. Robust classification (>90%) of high grade serous from all other ovarian tissue types was achieved. This is arguably the most important task as these tumors account for 70% of all ovarian malignancies and have the poorest survival rates. Moderately accurate classification was obtained for normal tissues relative to the other types. Interestingly, the least accuracy was obtained for LGS and benign tumors (Table 1). It has been suggested that the latter can evolve into LGS [5]; thus, if this supposition is correct, this poor discrimination would be expected.

Collectively the SHG and scattering metrics provided structural analyses of different aspects of structure for the different tissue types. These aspects are summarized in Table 2, where the readouts for classification are compared relative to the HGS tissues. For example, if only *μ*_*s*_^*′*^ values, which are primarily a measure of tissue density, were used to classify the tissues, both normal and HGS tissues were similar. However, with the inclusion of the *m* fit to the wavelength dependence, the F_SHG_/B_SHG_ emission directionality, and the relative SHG conversion efficiency, we note that the normal and HGS are discriminated, indicating they have very different ECM structural aspects. Using all these metrics and their structural meanings, not only greatly enhanced the classification of the tissues, but also helped to understand the importance of different physical features within the tumor microenvironment.

**Table 2:** Summary of independent metrics and their physical meaning relative to high-grade serous – Type II tissues

	*μ*_*s*_^′^	m shape value	F_SHG_/B_SHG_	Conversion efficiency
Normal	(≈) Density	1.32 (++) Cell and collagen structures more ordered	+ Disordered similar sized collagen fibrils	(- -) Less collagen organization at fiber level
Benign	(-) Density	1.01(- - -) Cell and collagen structures less ordered	(+ +) Large collagen fibrils	(-) Less collagen organization at fiber level
Endometrioid - Type I	(- -) Density	1.40 (+ + +) Cell and collagen structure more ordered	(+) Disordered similar sized collagen fibrils	(-) Decrease collagen density
Low-grade serous – Type I	(-) Density	1.41 (+ + +) Cell and collagen structure more ordered	(+ +) Larger more disordered collagen fibrils	(-) Less collagen organization at fiber level

(+) slightly increased, (++) moderately increased, (+++) highly increased, (≈) similar valued, (-) slightly decreased, (- -) moderately decreased, (- - -) highly decreased

In principle, all the metrics used here could be performed in conjunction with a laparoscope in a minimally invasive manner. For example, there already has been one report of microendoscopy for ovarian cancer [15]. In principle, such a design could be adapted to probe the SHG directionality (our work in progress). Currently, all women with suspected masses undergo oophorectomies even though these masses are frequently pathologically benign. However, SHG/optical scattering interrogation could potentially spare the ovaries if tumors were deemed to be benign. Moreover, the treatment course could be different if LGS versus HGS disease is found. More importantly, SHG/optical scattering could be used as a screening modality for early diagnosis of Type I and Type II ovarian cancer in postmenopausal women or high risk subjects (up to 30 fold) with a family history of this disease or known BRCA mutations.

## Conclusions

We have shown that SHG imaging microscopy and optical scattering measurements characterize the underlying ECM alterations in ovarian cancers and benign lesions relative to normal stroma. These approaches interrogate structures below the resolution of microscopy and provide collagen specific readouts. These quantitative methods are especially powerful when examining the wavelength dependencies as different tumor types have differing architectures relative to the interrogating spectrum. The ability to distinguish different classifications of ovarian cancer based on the collagen ECM is important for several reasons including: i) increased accuracy of clinical classification without the need of genomic analysis; ii) improve our understanding of the different etiologies of different ovarian cancers; and iii) potential to develop ovarian cancer type specific chemotherapeutics. This work provides the core data to show the potential of ECM collagen measurements as a new image-based biomarker for ovarian cancer. Future efforts will include further examination of collagen associated metrics, clinical trials to further show the linkage between collagen changes and clinical outcome, and the design of instrumentation that can exploit this information for clinical use.

## Abbreviations

ECM: Extracellular matrix
F_SHG_/B_SHG_: SHG directional emission ratio
g: Tissue anisotropy
HGS: High Grade Serous
LGS: Low Grade Serous
QPM: Quasiphasematching
SHG: Second Harmonic Generation
TEM: Transmission electron microscopy
λ_SHG_: SHG wavelength
μ_s_^′^: Reduced scattering coefficient
χ^(2)^: Nonlinear susceptibility tensor
